# Comparison of the Neutrophil-to-Lymphocyte and Platelet-to-Lymphocyte Ratios in Two Groups of Patients with Benign and Malignant Salivary Gland Tumors

**DOI:** 10.22038/ijorl.2025.89668.4001

**Published:** 2026

**Authors:** Mohammad Hossein Shafieyoun, Nezamoddin Berjis, Afrooz Eshaghian

**Affiliations:** 1 *Isfahan University of Medical Sciences, faculty of Medicine, Isfahan Iran. *; 2 *Department of Otorhinolaryngology, Head and Neck Surgery, Kashani Hospital, School of Medicine, Isfahan University of Medical Sciences, Isfahan Iran.*

**Keywords:** Neutrophil to lymphocyte ratio, Platelet to lymphocyte ratio, Benign masses, Malignant masses, Salivary glands

## Abstract

**Introduction:**

The ratio of neutrophils to peripheral lymphocytes is an inflammatory marker, and, based on previous studies, this ratio is associated with poor survival in several cancers. This study was conducted to compare the neutrophils-to-lymphocytes ratio (NLR) and platelets-to-lymphocytes ratio (PLR) in two groups of patients with benign and malignant salivary gland tumors.

**Materials and Methods:**

This study was cross-sectional, comparing two groups of patients with benign and malignant salivary gland masses. During the last 6 years (from 2018 to 2024), 380 Patients with a definite diagnosis of salivary gland tumors were included in the study, and NLR and PLR data were obtained from pre-operation laboratory exams.

**Results:**

NLR and PLR were significantly higher on average than in benign masses (P<0.05). The best cut point was 2.24 for NLR (sensitivity: 78%, specificity: 80%) and 104.35 for PLR (sensitivity: 68%, specificity: 60%).

**Conclusion:**

NLR and PLR are easy, practical methods that provide valuable information for diagnosing, assessing severity, and predicting prognosis of various diseases, such as salivary gland masses.

## Introduction

Salivary gland tumors (SGT) are complex and heterogeneous histologies that occur in the major glands (parotid, submandibular, and sublingual) and minor salivary glands (1). 80% of these tumors are benign but can recur or transform into malignant lesions. In 2005, the World Health Organization identified 24 malignant SGTs, the most prevalent of which were mucoepidermoid carcinoma, acinic cell carcinoma, and adenoid cystic carcinoma (2). 

In 2017, it identified 11 benign epithelial SGTs, the most common of which were pleomorphic adenoma, Warthin, tumor and myoepithelioma (3). Salivary gland malignancies account for 0.5–1.2% of all cancers and 5% of head and neck cancers (4). It affects women more often than men (1.5 to 1 ratio) and is more likely to be malignant in children (5). The risk of malignancy is also 15 to 32% for parotid masses, 41 to 50% for submandibular tumors, 70 to 90% for minor salivary glands, and almost 100% for sublingual masses (6). In minor salivary glands, 50% of tumors are malignant (the most often located in the palate) (7). The incidence rate of SGTs is 1.1 to 1.3 per 100,000 in the United States, compared with 1.31 per 100,000 in Europe and 1.04 per 100,000 in Japan (8). In a recent systematic review, for the Iranian population, the weighted mean prevalence of benign tumors was 66% and malignant tumors was 34% (9). The etiology of SGTs is poorly understood. Similar to other types of head and neck cancers, SGTs are associated with smoking and alcohol consumption (10). In addition, high consumption of processed meat, diets low in vegetables and high animal fat, obesity, and occupational radiation exposure are possible risk factors of salivary tumors. Epstein-Barr virus, immunodeficiency, and HIV infection have also been associated with an increased risk (11).

Some studies have suggested that neutrophils and platelets provide angiogenic, epithelial, and stromal growth factors that promote tumor progression. Several studies have also shown that C-reactive protein (CRP), albumin levels, neutrophil-to-lymphocyte ratio (NLR), and platelet-to-lymphocyte ratio (PLR) are associated with cancer stage (12). Interactions between the microenvironment and tumor cells play an important role in cancer progression. The microenvironment includes metabolic, inflammatory, and immune responses to stimuli from surrounding tissues (13). Recent basic studies have shown that tumor metastasis, microvascular remodeling, and tumor cell proliferation capabilities are influenced by the systemic inflammatory response (14,15). The increase of peripheral NLR (as an inflammatory marker) has been significantly associated with poor survival in several cancers, such as head and neck malignancies (16-18). 

Neutrophils are a subgroup of inflammatory cells that produce several factors, such as vascular endothelial growth factor, chemokines, and proteases, to facilitate angiogenesis; they can create a favorable tumor microenvironment that promotes cancer evolution (19). Neutrophils that infiltrate cancerous lesions can disrupt the immunity. In contrast, the number of lymphocytes existing in the tumor microenvironment can disturb the function of natural killer cells. In addition, increasing the number of lymphocytes reduces the levels of cytokines that can induce apoptosis in tumoral cells. Therefore, an increase in the neutrophil-to-lymphocyte ratio shows a poor prognosis for malignant SGTs (20). 

However, some of these associations remain unknown. The present study aimed to compare the NLR and PLR in two groups of patients with benign and malignant salivary gland masses over 6 years in Isfahan.

## Materials and Methods

The present study was a cross-sectional study comparing two groups of patients with benign and malignant SGTs, conducted in 2024. These patients had undergone surgery with a definitive diagnosis of the salivary gland or glands in two hospitals in Isfahan, Al-Zahra and Kashani, during the past 6 years (from 2018 to 2024). The inclusion criteria were patients aged 18 to 80 years with a pathology report of one or more salivary glands, and a complete blood count test was available in the medical file before the operation.

Patients who had other benign or malignant masses, were on dialysis, pregnant or breastfeeding, or those with inflammatory diseases, such as rheumatic arthritis, autoimmune, and infectious diseases, were not included in the study. After receiving the necessary and ethical permits (from the Faculty of Medicine, Isfahan University of Medical Sciences, No. IR.MUI.MED.REC.1403.136), the researcher reviewed the records of two hospitals in Isfahan, Al-Zahra and Kashani, and identified patients undergoing surgery with a definitive diagnosis of a salivary gland or glands during the past 6 years. After that, he reviewed the complete medical records and collected the required information using the prepared checklist. 

The checklist included the patient's demographic and clinical data, such as age, gender, pathology type, WBC, Neutrophil, Lymphocyte, and Platelet count on his record before surgery. Finally, 380 patients entered the study, and were divided into two groups: benign (322 patients) and malignant (58 patients). 

The collected data were analyzed using SPSS version 27 at a significance level of 0.05 with an independent T-test, Mann-whitney, and Chi-square tests, and ROC curve analysis. Sensitivity, specificity, and accuracy of the two main variables for predicting benign and malignant masses were measured using the Area Under the CURE (AUC) plot. Univariate and multivariate regression were also used to estimate the odds ratios.

## Results

All patients with a salivary gland tumor between 2018 and 2024 who had a documented CBC (complete blood count) and pathologic report at our tertiary hospital (Alzahra, Kashani) were included in the study. They excluded those without preoperative laboratory data or a documented pathologic report. Lab data were extracted from a standardized, frequently calibrated cell counter in our hospitals. Three hundred eighty patients entered the study; their demographic and medical characteristics are summarized in Table 1. 322 (84.7%) had benign masses, and 58 (15.3%) had malignant tumors. There was a significant difference in age between the two groups (P<0.05). In fact, the mean age of patients with malignant tumors was significantly higher than that of the other group.

 In both groups, there were more men than women, but no significant difference was observed (P>0.05).

**Table 1:** Demographic and clinical characteristics of the patients studied, divided into benign and malignant tumors

**Variables**	**Benign tumor**	**Malignant tumor**	**Total**	**p-value**
Age (mean ± SD)	51.43±16.22	67.43±17.61	55.19 ±17.61	<0.001
Sex distribution %				
Male	186 (57.8%)	38(65.5%)	224(58.9%)	0.269
Female	136(42.2%)	20 (34.5%)	156 (41.1%)	
NLR (mean ± SD)	1.9 ±1.08	4.3 ±3.94	2.25 ±1.99	<0.001
PLR (mean ± SD)	106.06 ±68.92	158.99 ±107.46	113.94 ±77.96	<0.001
Plt (mean ± SD)	232618.03 ±71230.39	202090.90 ±73559.65	228420.55 ±72171.16	0.024
WBC (mean ± SD)	8233.54 ±12384.26	7500 ±2579.36	8130.97 ±11524.05	0.0735

SD: standard deviation, NLR: neutrophil to lymphocyte ratio, PLR: platelet to lymphocyte ratio, PLt: platelet

NLR and PLR were significantly different between the two groups (P<0.05). In both ratios, malignant masses had a significantly higher mean than benign masses. Platelets also showed a significant difference between the two groups (P<0.05). Thus, malignant tumors had a significantly higher mean level than enign masses. In Table 2, NLR and PLR are shown by age and gender. In benign masses, NLR was not significantly different between the two age groups (less than and more than 52 years) (P>0.05) (52 years is the mean age of benign tumors). In malignant masses, the NLR did not differ significantly between the two age groups (less than and more than 67 years) (P>0.05) (67 years is the mean age of malignant tumors). 

**Table 2:** Neutrophil to Lymphocyte Ratio and Platelet to Lymphocyte Ratio by Age and Gender

**PLR**	**NLR**	** Variables**	
107.87 ± 22.87	1.94 ± 1.29	Male	Benign Mass
103.8±32.29	1.85 ± 0.73	Female	
0.675	0.556	P value
119.75 ± 165.65	5.02±4.47	Male	Malignant Mass
70.092 ± 142.65	0.85 ± 2.58	Female	
0.557	0.083	P value
109.18 ± 117.47	0.85 ±1.8	<= 52 years	Benign Mass
32.2 ± 94.48	0.66 ± 1.72	More than 52 years	
0.107	0.579	P value
100.9 ± 172.57	4.87 ± 4.09	<= 67 years	Malignant Mass
113.28 ± 149.11	3.2 ± 4.48	More than 67 years	
0.514	0.772	P value

Also, in this study, using the obtained data and statistical analyses, such as ROC (Receiver Operating Characteristic), thresholds were determined for each of the NLR and PLR variables to diagnose benign and malignant masses, which are commonly used in medical diagnostics to evaluate the performance of diagnostic tests.

**Fig 1:**
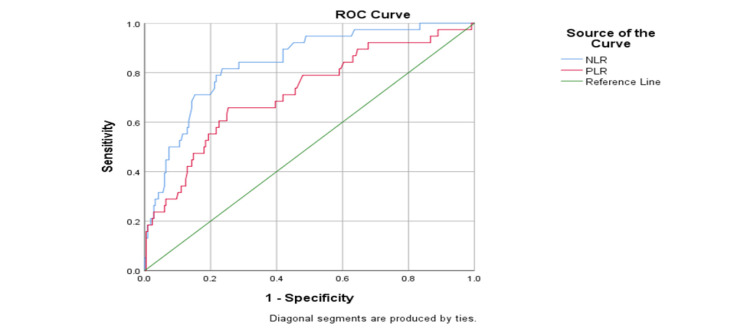
sensitivity and specificity of PLR (Platelet to neutrophil ratio and NLR (neutrophil to lymphocyte ratio)

The NLR threshold value is 2.24, Area under the curve (AUC) for NLR is 0.841 (CI95%: 0.775-0.907), which corresponds to the performance criteria of sensitivity: 78% and specificity: 80%, which means that using the NLR value more than 2.24, the test can correctly identify malignancy with 78% of positive cases (sensitivity) and 80% of negative cases (specificity).

The PLR ​​threshold value is 104.35, AUC for PLR is 0.720 (95%CI: 0.626-0.813), with the following performance criteria: sensitivity: 68% and specificity: 60%, indicates that using a PLR value of more than 104.35 as the threshold, the test can correctly identify 68% of positive cases (sensitivity) and 60% of negative cases (specificity) of malignant tumors. (Figure1).

These results can be used to evaluate the diagnostic accuracy of NLR and PLR in detecting malignancy. The NLR test appears to perform better overall, with higher AUC, sensitivity, and specificity than the PLR ​​test.

Based on significant univariate logistic regression results for NLR and PLR variables, they were included in a multivariate logistic regression model to predict benign and malignant masses. After performing multivariate logistic regression, only a significant increase in NLR was associated with malignant masses, with a 5-fold odds ratio (Table 3).

**Table 3:** Unavailable and Multivariate logistic regression analysis between variables

**Multivariate Logistic Regression Analysis**		**Unavailable Logistic Regression Analysis**		**Factors**
**P value**	**OR (95% CI)**	**P value**	**OR (95% CI)**	
0.042	5.339 (0.983-29.008)	0.001	1.405 (1.152-1.713)	NLR
0.020	1.070 (1.011-1.132)	0.0001	0.982 (0.977-0.987)	PLR

## Discussion

Recent investigations into the immune system have provided valuable insight into the potential impact of the immune response on tumor progression (21,22). Consequently, the significance of immunological biomarkers in cancer treatment and prognostic assessments has been highlighted. The role of the tumor microenvironment in tumor development has also been well recognized. One critical component is the inflammatory microenvironment, in which cells such as neutrophils and lymphocytes play a significant role in tumor progression (23). 

Several studies have focused on the importance of the neutrophil-to-lymphocyte ratio as a predictive biomarker for cancer. Kuzucu in Turkey divided and compared patients into two groups based on histopathological findings: patients with malignant or benign parotid tumors (n=145) and a control group (n=83) consisting of healthy individuals. The mean NLR in the malignant, benign, and healthy control groups was 2.51, 2.01, and 1.79, respectively, which were statistically significant differences. The PLR value of the malignant group was also significantly higher than the other two groups (benign, control) (23). Some studies failed to demonstrate PLR’s prognostic value in SGTs (24). In the present study, the mean NLR and PLR were much higher in the malignant mass group, and the optimal PLR cut point was 104.25. In Magdum’s study of 50 patients, an NLR of 4 or greater was considered a good marker for the presence of oral cancer (Poor prognosis with higher NLR) (25). Cheng also studied 45 patients with primary parotid cancer in China. The mean NLR was 2.48 with a range of 1.5 to 6.1 and was significantly associated with tumor stage, disease stage, and disease grade. They reported in previous studies that the best cutoff value ranged from 1.98 to 5 for head and neck, breast, gynecologic, colorectal, and oral cavity cancers; however, the standard cutoff point remains unknown, and their best cutoff was 2.48 (26). Szilasi reviewed the clinical data of 156 head and neck squamous cell carcinoma patients for survival in Poland. The results indicated that a higher NLR could be associated with an increased risk of overall survival. Their optimal threshold was 107 for PLR and 3.9 for NLR (27). Lee studied 20 patients with salivary gland cancers treated with pembrolizumab in the United States. The results showed that a high pre-treatment NLR was independently associated with 6-month survival (28). The results of Bora’s study in Turkey also showed that the NLR can be used as a biomarker for submandibular gland masses and has prognostic significance in malignant masses (29). In the present study with 380 patients, the NLR was also significantly higher in malignant masses, and the optimal cut point for salivary gland malignancies was 2.24.

Damar also reported that the mean percentage of neutrophils in patients with malignant oral masses was significantly higher than in those with benign oral masses, consistent with the results of the present study (19). 

Committeri concluded, after studying 117 patients with SGTs in Italy, that inflammatory biomarkers and radiomic features can provide surgeons with faster information than differential diagnosis before surgery. Their analysis revealed the best cutoff points for separating a benign mass from a malignant one (PLR: 133.30; NLR: 3.62), with an accuracy of 0.88 for NLR (3).

Finally, it should be noted that the NLR and PLR are readily available, cost-effective, and reproducible inflammatory indicators for distinguishing patients with different SGTs from healthy individuals. PLR showed lower accuracy but may still be helpful when combined with NLR or the systemic immune-inflammation index (SII). NLR and PLR are not shown to be impacted by age and gender so that they can be used as promising biomarkers in the absence of prior biopsy. NLR can help limit biopsy decisions and early suspicion of malignancy, and it can help with prognosis.

The limitations of this study include the lack of patient follow-up, a single-region design, a retrospective design, and the absence of a healthy control group. Only 58 malignant cases versus 322 benign; this may affect predictive model robustness, but the prevalence of malignancy in salivary gland tumors is less than the benign number, and imbalance in group sizes is another one of our limitations.

It would also be better to compare these two indices in individuals with malignant and benign masses with healthy individuals, through prospective, multicenter validation and correlation with survival outcomes.

## Conclusion

The neutrophil-to-lymphocyte ratio and platelet-to-lymphocyte ratio are easy, practical methods that provide valuable information for diagnosing, assessing severity, and predicting prognosis in various diseases, including salivary gland masses. These results can be used to evaluate the diagnostic accuracy of NLR and PLR in detecting malignancy. The NLR test appears to perform a better overall, with higher sensitivity and specificity than the PLR ​​test.
